# MUC16：肿瘤治疗新靶点

**DOI:** 10.3779/j.issn.1009-3419.2022.101.31

**Published:** 2022-07-20

**Authors:** 茹云 高, 宁 娄, 晓红 韩, 远凯 石

**Affiliations:** 1 100021 北京，国家癌症中心，国家肿瘤临床医学研究中心，抗肿瘤分子靶向药物临床研究北京市重点实验室，中国医学科学院北京协和医学院肿瘤医院内科 Department of Medical Oncology, National Cancer Center, National Clinical Research Center for Cancer, Cancer Hospital, Chinese Academy of Medical Sciences & Peking Union Medical College, Beijing Key Laboratory of Clinical Study on Anticancer Molecular Targeted Drugs, Beijing 100021, China; 2 100021 北京，国家癌症中心，国家肿瘤临床医学研究中心，抗肿瘤分子靶向药物临床研究北京市重点实验室，中国医学科学院北京协和医学院肿瘤医院检验科 Department of Clinical Laboratory, National Cancer Center, National Clinical Research Center for Cancer, Cancer Hospital, Chinese Academy of Medical Sciences & Peking Union Medical College, Beijing Key Laboratory of Clinical Study on Anticancer Molecular Targeted Drugs, Beijing 100021, China; 3 100730 北京，中国医学科学院北京协和医院临床药理研究中心，疑难重症及罕见病国家重点实验室，国家药监局药物临床研究与评价重点实验室，创新药物临床PK/PD北京市重点实验室 Clinical Pharmacology Research Center, Peking Union Medical College Hospital, State Key Laboratory of Complex Severe and Rare Diseases, NMPA Key Laboratory for Clinical Research and Evaluation of Drug, Beijing Key Laboratory of Clinical PK & PD Investigation for Innovative Drugs, Chinese Academy of Medical Sciences & Peking Union Medical College, Beijing 100730, China

**Keywords:** MUC16, 肿瘤治疗, 粘蛋白, 免疫治疗, 靶向治疗, MUC16, Tumor therapy, Mucin, Immunotherapy, Targeted therapy

## Abstract

黏蛋白16（mucin16, MUC16），又称癌抗原125（carbohydrate antigen 125, CA125），是1981年美国科学家Bast等从卵巢上皮癌抗原中检测出且能被单克隆抗体OC125识别的一种糖蛋白抗原。CA125在正常卵巢组织中不存在，但在卵巢上皮癌患者的血清中常见升高。CA125是卵巢上皮癌诊断和监测复发最常用的血清学生物标志物。MUC16在多种肿瘤中高表达，可以与半乳糖凝集素-1/3（galectin-1/3）、间皮素、唾液酸结合的免疫球蛋白样凝集素-9（sialic acid-binding immunoglobulin-type lectins-9, Siglec-9）等配体结合，通过多种肿瘤相关信号通路，对肿瘤发生发展、迁移侵袭及肿瘤免疫具有重要作用。靶向MUC16的治疗方式已经取得一定成效，相关临床前研究以及临床试验正在进行当中，MUC16可能是一个有潜力的肿瘤治疗新靶点。本文重点阐述MUC16在肿瘤发生发展中的作用，关注其在肿瘤治疗中的研究现状，为后续靶向MUC16的肿瘤治疗研究提供参考。

## Abstract (en)

Mucin16 (MUC16), also known as carbohydrate antigen 125 (CA125), is a glycoprotein antigen that can be recognized by the monoclonal antibody OC125 detected from epithelial ovarian carcinoma antigen by Bast *et al* in 1981. CA125 is not present in normal ovarian tissue but is usually elevated in the serum of epithelial ovarian carcinoma patients. CA125 is the most commonly used serologic biomarker for the diagnosis and recurrence monitoring of epithelial ovarian carcinoma. MUC16 is highly expressed in varieties of tumors. MUC16 can interact with galectin-1/3, mesothelin, sialic acid-binding immunoglobulin-type lectins-9 (Siglec-9), and other ligands. MUC16 plays an important role in tumor genesis, proliferation, migration, invasion, and tumor immunity through various signaling pathways. Besides, therapies targeting MUC16 have some significant achievements. Related preclinical studies and clinical trials are in progress. MUC16 may be a potential novel target for tumor therapy. This article will review the mechanism of MUC16 in tumor genesis and progression, and focus on the research actuality of MUC16 in tumor therapy. This article also provides references for subsequent tumor therapy studies targeting MUC16.

粘蛋白16（mucin16, MUC16），又称癌抗原125（carbohydrate antigen 125, CA125）。Bast等^[1]^于1981年发现一种单克隆抗体OC125能与卵巢上皮癌细胞反应，但是与正常组织不反应，能与这种抗体反应的抗原被称为CA125。2001年，Yin等^[2]^鉴定CA125的克隆结构属于MUC家族，将其命名为MUC16。自发现以来，CA125一直是卵巢癌临床诊断和监测复发的重要血清学标志物。然而，由于生理状态（如怀孕前3个月），非恶性疾病（如子宫内膜异位症和卵巢囊肿等妇科良性疾病）和其他非妇科恶性肿瘤也会导致血清CA125升高，CA125在卵巢癌的诊断和疗效监测中特异度较低^[3]^。研究发现，MUC16与多种肿瘤信号通路相关，在肿瘤免疫微环境中也起到重要作用，靶向MUC16的治疗方式逐渐成为一个研究热点。本文重点阐述MUC16在肿瘤发生发展中的作用，关注其在肿瘤治疗中的研究现状，为后续靶向MUC16的肿瘤治疗研究提供参考。

## 1 MUC16结构与功能概述

MUC家族是一类由上皮细胞产生的，大分子量、高度糖基化的蛋白家族，以串联重复氨基酸序列为特征^[4]^，具有润滑、参与细胞信号转导及形成化学屏障的功能。人类MUC家族包含MUC1-MUC21，可以分为两种类型：①分泌型：包括MUC2、MUC6、MUC19等；②跨膜型：具有高度糖基化的胞外域、跨膜域和短的胞质尾端，包括MUC1、MUC4、MUC16等^[4]^。分泌型粘蛋白可以形成物理屏障，作为一种黏液凝胶，为呼吸道和胃肠道上皮细胞提供保护，并构成肝脏、乳房、胰腺和肾脏等器官的导管表面；跨膜型粘蛋白通过它们胞外域的O糖基化串联重复序列形成棒状结构，构建保护性的黏液凝胶层，黏液凝胶层可以扩展到细胞表面100 nm以外，并且有大于10 nm的多糖萼^[5]^。

*MUC16*基因位于人类染色体19p13.2上，由179 kb的基因组DNA编码。它是迄今为止发现的最大的粘蛋白，相对分子量为2, 000 kDa-5, 000 kDa。MUC16是一种I型跨膜蛋白，主要包含三个部分：N端结构域，串联重复结构域（tandem repeat, TR）和C端结构域。N端结构域长度约12, 000个氨基酸；TR结构域包含18个-60个可变数目的串联重复序列，每个重复序列有156个氨基酸，包含56个海胆精子蛋白-肠激酶-聚集蛋白（sea urchin sperm, enterokinase and agrin, SEA）结构域以及两个锚蛋白域；C端结构域由284个氨基酸组成，又可以分为细胞外部分（包含假定的裂解位点）、单次跨膜结构域，细胞质尾端[包含埃兹蛋白/根蛋白/膜突蛋白（ezrin/radixin/moesin, ERM）结合域和推定的核定位信号RRRKK]^[3, 4, 6-9]^。MUC16主要存在两种糖基化形式：N-糖基化和O-糖基化，N端结构域主要以O-糖基化为主，TR区域包含N-糖基化和O-糖基化，其丰富的丝氨酸和苏氨酸残基可能是糖基化的潜在位点^[4, 6]^。MUC16常表达于眼表的角膜和结膜、呼吸道和女性生殖道黏膜上皮等部位，在水化、润滑上皮，作为屏障保护细胞免受侵害等方面发挥重要作用^[10, 11]^。

## 2 MUC16在肿瘤发生发展中的作用

MUC16在包括卵巢癌^[1]^、乳腺癌^[12]^、宫颈癌^[13]^、胰腺癌^[14]^、结直肠癌^[15]^在内的多种肿瘤中表达异常升高，这种高表达与癌症进展、转移以及患者的不良预后相关^[14, 16]^。MUC16可以与多种肿瘤相关信号通路相互作用，能促进肿瘤细胞增殖、肿瘤迁移侵袭，表达MUC16的肿瘤细胞还可以通过与多种免疫细胞相互作用从而发生免疫逃逸。此外，MUC16高频突变与肿瘤新生抗原形成、免疫细胞浸润及免疫检查点抑制剂（immune checkpoint inhibitors, ICIs）疗效相关，有望成为预测免疫治疗疗效的生物标志物。

### 2.1 MUC16相关配体

在肿瘤研究中，MUC16相关配体主要有以下几种：①半乳糖凝集素-1/3（galectin-1/3）：MUC16的核心N-糖基化和O-糖基化位点能与galectin家族的galectin-1^[17]^和galectin-3^[18]^结合，促进肿瘤细胞增殖；②间皮素：MUC16与间皮素结合的亲和力较高，可介导细胞粘附，促进肿瘤转移，这种结合也依赖于N-糖基化位点^[19, 20]^；③唾液酸结合的免疫球蛋白样凝集素-9（sialic acid-binding immunoglobulin-type lectins-9, Siglec-9）：MUC16可以结合表达在多种免疫细胞表面的抑制性受体Siglec-9，阻断肿瘤细胞和免疫细胞的相互作用^[21]^；④选择素：研究^[22, 23]^发现MUC16是L-选择素和E-选择素的配体，可能与肿瘤转移侵袭相关。

### 2.2 MUC16与肿瘤增殖

#### 2.2.1 JAK2/STAT3信号通路

Janus激酶2（Janus kinase 2, JAK2）/信号转导及转录激活因子3（signal transducer and activator of transcription 3, STAT3）信号通路是MUC16促进肿瘤生长增殖的一个重要通路。Batra的团队^[12]^通过免疫共沉淀（co-immunoprecipitation, Co-IP）验证了MUC16和JAK2之间的相互作用，并发现其可以通过激活STAT3和c-Jun介导乳腺癌增殖，并通过抑制肿瘤坏死因子相关的凋亡诱导配体（tumour necrosis factor related apoptosis inducing ligand, TRAIL）途径抑制乳腺癌细胞的凋亡。MUC16通过JAK2/STAT3通路促进肿瘤增殖在宫颈癌、结直肠癌、胰腺癌和肺癌等肿瘤中也有报道^[13, 15, 24, 25]^。

#### 2.2.2 EGFR-PI3K-Akt信号通路

MUC16可以通过直接或间接方式激活表皮生长因子受体（epidermal growth factor receptor, EGFR）-磷脂酰肌醇3激酶（phosphatidylinositol3 kinase, PI3K）-蛋白激酶B（protein kinase B, PKB/Akt）信号通路促进肿瘤增殖。Rao等^[18]^运用免疫荧光定位和三重免疫沉淀的方法发现MUC16可以与EGFR以及整合素β1相互作用，且这一过程需要O-6-甲基鸟嘌呤-DNA甲基转移酶（O6-methylguanine-DNA methyltransferase, MGMT）依赖的N-糖基化和MUC16的配体galectin-3参与。Stasenko等^[26]^进一步发现，在MUC16高表达的肿瘤中，靶向galectin-3的抗体可以阻断Akt和细胞外调节蛋白激酶（extracellular regulated protein kinases, ERK）1/2磷酸化，进而抑制乳腺癌生长。Thomas等^[14]^在胰腺癌研究中发现，MUC16可以直接与EGFR相互作用激活肿瘤细胞中Akt和糖原合酶激酶3β（glycogen synthase kinase, GSK3β）致癌信号通路，增强恶性程度，促进胰腺癌向侵袭性表型发展。

#### 2.2.3 肿瘤代谢相关信号通路

MUC16也可以通过调节肿瘤代谢途径促进肿瘤增殖，这可能与肿瘤细胞的瓦氏效应（Warburg effect）相关。Shukla等^[27]^在胰腺癌的研究中发现，MUC16可以诱导肿瘤细胞的代谢重编程，增加糖酵解，增强肿瘤活性和侵袭性，MUC16有可能通过哺乳动物雷帕霉素靶蛋白（mammalian target of rapamycin, mTOR）及其下游靶点细胞性骨髓细胞瘤病病毒癌基因（cellular myelocytomatosis viral oncogene, c-MYC）来调控葡萄糖转运蛋白1（glucose transporter 1, *GLUT1*）以及己糖激酶II（hexokinase II, *HK II*）等基因表达，进而调控代谢。Wang等^[28]^在卵巢癌研究中进一步验证，MUC16可以基于对GLUT1表达的调控，来控制葡萄糖摄取，进而促进肿瘤增殖。Fan等^[29]^也在胆囊癌的研究中发现，MUC16的C末端可以与果糖二磷酸醛缩酶C（aldolase C, ALDOC）结合，破坏ALDOC对葡萄糖缺乏的感知能力，进而促进胆囊癌细胞的葡萄糖摄取和糖酵解，促进肿瘤细胞增殖。

### 2.3 MUC16与肿瘤迁移侵袭

肿瘤中MUC16高表达能促进迁移侵袭。Miyajima团队^[20]^在卵巢癌研究中发现，MUC16可以通过与间皮素结合使肿瘤细胞粘附到间皮细胞上，进而促进卵巢癌的腹膜转移。Patankar团队^[19]^进一步发现这一过程需要N-糖基化位点的参与。之后，Chen等^[30]^发现MUC16与间皮素的相互作用，可以通过激活基质金属蛋白酶-7（matrix metalloproteinase-7, MMP-7）来促进肿瘤细胞的迁移和侵袭。在胰腺癌的研究中，Batra团队^[31]^通过Co-IP发现，MUC16还可以通过局部粘着斑激酶（focal adhesion kinase, FAK）介导的信号通路促进胰腺癌的转移。Wnt/β-catenin信号通路也是MUC16促进肿瘤转移的信号机制之一。Liu等^[32]^发现MUC16的C末端结构域可以通过与β-catenin相互作用激活Wnt/β-catenin信号通路，促进肿瘤的发生和转移。Giannakouros等^[33]^发现，MUC16的C末端可以通过抑制β-catenin的降解促进E-cadherin介导的肿瘤细胞团簇形成，在卵巢癌进展中发挥关键作用。此外，Chen等^[23]^发现MUC16是L-选择素和E-选择素的配体，选择素家族通过介导肿瘤细胞上的选择素配体与微血管中表达选择素的内皮细胞相互作用促进肿瘤转移，选择素途径也可能是MUC16参与肿瘤转移的方式。

### 2.4 MUC16与肿瘤免疫

MUC16对肿瘤免疫具有双重作用。一方面，MUC16可以通过与自然杀伤（natural killer, NK）细胞、巨噬细胞等免疫细胞表面的抑制性受体结合，发挥抑制肿瘤免疫的作用；另一方面，MUC16促进树突状细胞（dendritic cell, DC）成熟，MUC16突变产生的新生抗原能激活CD8^+^T细胞，并富集多种肿瘤浸润免疫细胞，发挥促进肿瘤免疫的作用。

MCU16对肿瘤免疫的抑制作用主要表现在对固有免疫细胞的作用。Patankar团队^[34]^发现MUC16对NK细胞有较强的抑制作用，MUC16敲除的卵巢癌细胞更容易被NK细胞裂解，在NK细胞白血病细胞系（NK cell leukemia cell line, NKL）刺激下存活下来的肿瘤细胞表达更高水平的MUC16，这表明NKL选择性裂解了MUC16低表达的肿瘤细胞^[35]^。之后他们进一步发现，MUC16可以通过结合表达在B细胞、单核细胞及NK细胞等多种免疫细胞表面的抑制性受体Siglec-9，促进肿瘤免疫逃逸^[21]^。除了能抑制NK细胞的细胞毒效应以及阻断NK细胞与肿瘤细胞的结合外，MUC16对巨噬细胞功能也有相应的抑制作用^[36]^。

*MUC16*突变产生的新生抗原在肿瘤免疫中有着重要作用，这可能预示着MUC16可以作为基于肿瘤新抗原的个体化疫苗的潜在靶点。*MUC16*在泛肿瘤中突变频繁，是突变频率位居前三的基因之一^[37]^，但由于其体积较大，常被排除在显著突变基因列表之外^[38]^。2017年，在一项针对胰腺癌长期存活者（long-term survivor, LTS）的研究中，Balachandran等^[39]^发现*MUC16*突变产生的新生抗原是LTS长期存活的原因。他们发现LTS主要与高质量新生抗原的产生以及肿瘤CD8^+^T细胞高浸润有关，同时他们发现这些独特的新抗原具有富集并激活CD8^+^T细胞的能力，进而鉴定出*MUC16*是与LTS相关的独特新抗原优先富集的位点，他们将其称之为“热点突变”。此后，*MUC16*高突变与免疫细胞、免疫相关通路以及ICIs疗效之间的相关性渐渐在多项肿瘤的突变分析中被证实。*MUC16*高突变与肿瘤免疫相关性在黑色素瘤^[40]^、胃癌^[41]^、肝癌^[42]^、结肠癌^[43]^、宫颈癌^[44]^、子宫内膜癌^[45]^等多种肿瘤的基因组突变分析中均有报道。Zhang等^[46]^在一项涉及30个实体瘤，涵盖了9, 850个样本的mRNA表达谱的泛癌分析中发现，*MUC16*突变的肿瘤微环境中有更丰富的免疫细胞浸润，GSEA富集分析也证明了*MUC16*突变主要富集在免疫相关通路中，且*MUC16*的高突变预示了一个良好的ICIs治疗应答，有望成为预测免疫治疗疗效的生物标志物。在另一项针对卵巢癌的研究中，Zhai等^[47]^发现MUC16可以促进T细胞向CD8^+^T细胞转化，并且显著上调了DC的成熟分子CD80、CD83和CD86，这可能为基于DC的免疫疗法应用于临床提供依据。

## 3 靶向MUC16的治疗方式

MUC16在多种肿瘤中高表达，并且在肿瘤相关信号通路中有着显著作用，是一个有潜力的肿瘤治疗靶点。MUC16作为跨膜粘蛋白，其细胞外结构域可能成为抗体介导的治疗方式的良好靶点，比如单克隆抗体、抗体偶联药物（antibody-drug conjugate, ADC）、嵌合抗原受体（chimeric antigen receptors, CARs）和双特异性T细胞衔接体（bi-specific T-cell engagers, BiTEs）等。MUC16在肿瘤免疫中的作用也提示了开发基于MUC16的个体化肿瘤新抗原疫苗的可能性。下面将详细阐述目前靶向MUC16的治疗方式的相关研究。

### 3.1 单克隆抗体

Oregovomab（OvaRex）是MUC16第一个应用于临床试验的单克隆抗体，其活性成分是鼠单克隆抗体mAb B43.13。mAb B43.13对CA125具有非常高的亲和力（1.16×10^10^/m），虽然Oregovomab单药治疗未能显示出显著的临床收益^[48, 49]^，但Oregovomab联合治疗取得了有希望的结果。针对97例晚期卵巢癌患者的一项Ⅱ期临床试验^[50]^结果显示，一线应用卡铂-紫杉醇化疗联合Oregovomab（carboplatin-paclitaxel chemotherapy plus four immunizations with oregovomab, CPO）比起单纯卡铂-紫杉醇化疗（carboplatin-paclitaxel chemotherapy, CP），无进展生存期（progression-free survival, PFS）有着显著改善，分别为41.8个月（CPO）*vs* 12.2个月（CP）（*P*=0.002, 7, HR=0.46, 95%CI: 0.28-0.70）。另一项Oregovomab联合卡铂-紫杉醇化疗的Ⅲ期临床试验正在进行之中（ClinicalTrials.gov Identifier: NCT04498117），Oregovomab联合化疗或可为晚期卵巢癌患者提供新的治疗选择。

MUC16的糖基化位点逐渐成为癌症中一个有潜力的生物标志物和靶向治疗的位点。Olson等^[51]^研究发现，一种靶向MUC16糖基化位点的单克隆抗体mAb-AR-9.6，可以特异性结合MUC16的SEA结构域5，抑制人类表皮生长因子受体2（human epidermal growth factor receptor 2, HER2）型受体的磷酸化和下游AKT/GSK3β信号通路，进而抑制肿瘤的致瘤活性和肿瘤生长^[14]^，AR-9.6和MUC16的结合能力也在卵巢癌和胰腺癌细胞的体外实验以及人源肿瘤组织来源移植瘤（patient-derived xenograft, PDX）小鼠模型体内实验^[52]^中得到了验证，AR9.6有望成为一种新的抗MUC16单抗药物。

### 3.2 ADC

ADC包含单克隆抗体、连接子、细胞毒性药物三部分，可以利用单克隆抗体的靶向特异性将细胞毒性药物运送到表达相应抗原的肿瘤细胞所在的位置，达到治疗目的。美国基因泰克公司开展了两项针对MUC16的ADC的Ⅰ期临床试验，RG-7458（DMUC-5754A）（ClinicalTrials.gov Identifier: NCT01335958）和RG-7882（DMUC-4064A）（ClinicalTrials.gov Identifier: NCT02146313）^[53, 54]^，这两项研究针对铂类耐药的卵巢癌和不可切除的胰腺癌。研究结果显示DMUC-5754A和DMUC-4064A具有可接受的安全性。DMUC-5754A应答率为17%（5/29），包括1例完全缓解（complete response, CR）、4例部分缓解（partial response, PR）^[53]^。DMUC-4064A总临床获益率为42%（27/65），包括CR、PR和疾病稳定（stable disease, SD）超过6个月的患者^[54]^。如何在保证安全性的基础上提高疗效是未来需要进一步解决的问题。

### 3.3 CARs

CAR包含可以特异性识别肿瘤相关抗原（tumor-associated antigen, TAA）的单链抗体、跨膜结构域和T细胞受体（T cell receptor, TCR）的胞内激活结构域，CAR能够在不受主要组织相容性复合体（major histocompatibility complex, MHC）限制的情况下直接激活T细胞。早在2010年，Brentjens团队^[55]^就通过靶向MUC16抗原的嵌合抗原受体T细胞（chimeric antigen receptor T-cell, CAR-T）疗法，成功根除小鼠卵巢癌。为了解决CAR-T细胞被肿瘤微环境抑制的问题，他们进一步开发了一种能共表达白细胞介素12（interleukin 12, IL-12）的靶向MUC16表位的CAR-T，并在体内实验中取得了良好的抗肿瘤效果^[56]^。目前，这种CAR-T疗法治疗MUC16表位阳性的复发性实体瘤的Ⅰ期临床试验正在进行当中（ClinicalTrials.gov identifier: NCT02498912）。此外，Li^[57]^在卵巢癌研究中发现，靶向细胞程序性死亡配体1（programmed cell death 1 ligand 1, PD-L1）和MUC16抗原的双CAR-T细胞疗法，与单独靶向PD-L1或MU16的单CAR-T细胞疗法相比疗效提高了2倍-4倍。但CAT-T细胞疗法仍存在脱靶、安全性不高等问题，其在实体瘤中的应用也有所限制，有待于进一步的研究。

### 3.4 BiTEs

BiTEs是一种以T细胞作为效应细胞的双特异性单链抗体，BiTEs具有两个抗原结合部位，可以同时和T细胞表面的TCR复合物（一般为CD3）及肿瘤细胞表面的特定TAA结合^[58]^，从而有效地激活效应T细胞来达到杀伤肿瘤细胞的目的。目前靶向MUC16的BiTEs已经取得较好的抗肿瘤疗效，且毒副作用较低，是一种有潜力的联合抗肿瘤治疗方式。美国再生元制药公司（Regeneron Pharmaceuticals）开发的靶向MUC16的BiTEs-REGN4018（MUC16/CD3 BiTE），能够同时靶向MUC16阳性的肿瘤细胞和表达CD3的T细胞，诱导多克隆T细胞激活。REGN4018在人源化的小鼠模型和食蟹猴模型中展现出良好的抗肿瘤能力和较小的毒性反应，其与抗程序性死亡受体1（programmed cell death 1, PD-1）抗体联用可以增强疗效^[59]^，目前REGN4018单独或联合PD-1单抗治疗复发卵巢癌患者的Ⅱ期临床试验正在进行当中（ClinicalTrials.gov Identifier: NCT03564340）。该公司的另一种靶向MUC16的BiTEs-REGN-5668（MUC16/CD28）联合REGN4018或PD-1单抗治疗复发卵巢癌的研究也已经进入Ⅱ期临床试验（ClinicalTrials.gov Identifier: NCT04590326）。此外，Yeku等^[60]^的研究表明，MUC16/CD3 BiTE联合抗PD-1或抗血管内皮生长因子（vascular endothlial growth factor, VEGF）均可增强疗效，但联合抗VEGF优于联合抗PD-1，相关结果有待于进一步临床试验的证实。

### 3.5 肿瘤疫苗

肿瘤疫苗是一种利用肿瘤抗原激活机体免疫系统，产生抗肿瘤免疫反应以控制或清除肿瘤的一种肿瘤免疫治疗方法，主要分为：全细胞疫苗、多肽疫苗和核酸疫苗。MUC家族的MUC1是一种粘蛋白样、糖基化的TAA，是设计和开发肿瘤疫苗的热门靶点之一，多项MUC1为靶点的肿瘤疫苗已经进入临床试验阶段，但临床试验效果不一^[61]^。部分MUC1疫苗没有产生临床有效的抗肿瘤免疫效应，可能是因为TAA特异性T细胞受中枢和/或外周耐受的限制，TAA不能有效激活T细胞^[62]^。近年来，基于肿瘤新抗原的个体化肿瘤疫苗，由于特异性强、安全性高，已经成为精准医疗下的研究热点。一项针对手术切除后的Ⅲ期或Ⅳ期黑色素瘤患者的临床试验（ClinicalTrials.gov Identifier: NCT01970358）^[63]^显示，在6例接种个体化肿瘤新抗原疫苗的患者中，4例患者接种后25个月没有复发，2例复发患者在接受了抗PD-1治疗后肿瘤完全消退，且观察到患者新抗原特异性T细胞库的扩增。另一项针对胶质母细胞瘤的临床试验（ClinicalTrials.gov Identifier: NCT02287428）^[64]^显示个体化肿瘤新抗原疫苗可以改善低突变负荷和免疫“冷肿瘤”患者的免疫微环境。MUC16在泛肿瘤中高频突变，是与长期生存相关的高质量新生抗原的富集位点，能促进CD8^+^T细胞的富集和激活^[39]^，且与DC的成熟密切相关^[47]^，MUC16有望成为基于肿瘤新抗原的个体化疫苗的新靶点。

## 4 总结与展望

高度糖基化的粘蛋白MUC16在多种肿瘤中高表达，越来越多的研究表明MUC16在多种肿瘤相关信号通路中起到重要作用，但MUC16参与肿瘤发生发展过程的具体分子机制仍不明确，基于相关信号通路的药物开发有待于进一步研究。目前靶向MUC16胞外表位的抗体介导的治疗方式已经取得一定成效，但还存在疗效不佳、安全性不高等问题，联合其他药物可能是较好的治疗策略。*MUC16*高突变产生的新生抗原在肿瘤免疫中有着重要作用，针对MUC16新生抗原的个体化肿瘤疫苗及其他免疫疗法可能成为一个新的研究方向。
